# Alternative trait combinations and secondary resource partitioning in sexually selected color polymorphism

**DOI:** 10.1002/ece3.610

**Published:** 2013-05-31

**Authors:** Yuma Takahashi, Masakado Kawata

**Affiliations:** Division of Ecology and Evolutionary Biology, Graduate School of Life Sciences, Tohoku UniversityAoba, Sendai, Miyagi, 890-8578, Japan

**Keywords:** Body color, camouflage, choice, damselfly, egg morphology, female polymorphism, oviposition, phenotypic integration, resource partitioning

## Abstract

Resource partitioning within a species, trophic polymorphism is hypothesized to evolve by disruptive selection when intraspecific competition for certain resources is severe. However, in this study, we reported the secondary partitioning of oviposition resources without resource competition in the damselfly *Ischnura senegalensis*. In this species, females show color polymorphism that has been evolved as counteradaptation against sexual conflict. One of the female morphs is a blue-green (andromorph, male-like morph), whereas the other morph is brown (gynomorph). These female morphs showed alternative preferences for oviposition resources (plant tissues); andromorphs used fresh (greenish) plant tissues, whereas gynomorphs used decaying (brownish) plants tissues, suggesting that they chose oviposition resources on which they are more cryptic. In addition, the two-color morphs had different egg morphologies. Andromorphs have smaller and more elongated eggs, which seemed to adapt to hard substrates compared with those of gynomorphs. The resource partitioning in this species is achieved by morphological and behavioral differences between the color morphs that allow them to effectively exploit different resources. Resource partitioning in this system may be a by-product of phenotypic integration with body color that has been sexually selected, suggesting an overlooked mechanism of the evolution of resource partitioning. Finally, we discuss the evolutionary and ecological consequences of such resource partitioning.

## Introduction

Two or more coexisting species that utilize similar resources often demonstrate resource partitioning, and this partitioning has been hypothesized to reduce competition between the species (Pyke 1982; Grant and Grant 2006). Resource partitioning, known as trophic polymorphism, is also found within a species (Skúlason and Smith 1995; McLaughlin et al. 1999). Sympatric trophic polymorphisms are suggested to evolve by disruptive selection when intraspecific competition for certain resources is severe (Maynard-Smith 1962; Hendry et al. 2009). Such partitioning for resources, including the habitat, can lead to some interesting evolutionary and ecological consequences (Rueffler et al. 2006). Trophic polymorphism constitutes an intermediate stage in the formation of new species, leading to sympatric speciation (Skúlason and Smith 1995). In addition, resource partitioning has been predicted to ease the intense intraspecific competition for resources similar to interspecific resource partitioning (McLaughlin et al. 1999; Forsman et al. 2008), which affects population and community dynamics.

When resource partitioning evolves within a species, correlational selection, which favors alternative adaptive trait combinations, promotes the physical integration and association between traits to improve resource partitioning (Brodie 1989; Forsman et al. 2002; Ahnesjö and Forsman 2003). Two morphs in the Neotropical cichlid *Cichlasoma citrinellum* feed on different prey, that is, soft snails and hard snails (Meyer 1989), and the morphs are distinguished on the basis of morphological traits (Meyer 1989; Ruzzante et al. 1998). In other cases, color polymorphisms evolve in trophic polymorphisms because the alternative resource use makes a difference in biotic and abiotic environments between morphs, especially background color and ambient light conditions (Nosil 2004; Maan and Seehausen 2011). In walking sticks, two morphs use different plants species as food and their color pattern correspond to each plant species (Sandoval 1994a,b; Nosil 2004). The diversity of resource use can lead to color and/or pattern polymorphisms as a by-product of phenotypic integration.

However, in some organisms the diversity in color seems to evolve independent of resource partitioning. Color polymorphisms relating to warning color and Müllerian and Batesian mimics are not considered to be the by-products of evolution of resource partitioning because the primary role of these color polymorphisms is to avoid the risks of predation (Brown and Benson 1974; Darst and Cummings 2006; Nokelainen et al. 2012). Other examples are sex-limited color polymorphisms, which are suggested to evolve throughout sexual selection (Svensson et al. 2008). When color polymorphism is limited to males, it mostly plays a role in male–male competition for access to females (Tsubaki et al. 1997; Plaistow et al. 2000). On the other hand, female-limited polymorphisms are important for regulating costly intersexual interactions (Cook et al. 1994; Svensson et al. 2005; Takahashi et al. 2010). Female damselflies exhibit color polymorphisms, consisting of ancestral females (gynomorphs) and male-like morphs (andromorphs) (Corbet 1999; Gosden and Svensson 2009). Male-like color patterns are suggested to evolve to avoid male mating harassment, suggesting that the color polymorphisms in female damselflies are an evolutionary response against selection pressure derived from male harassment (Robertson 1985).

Resource partitioning could evolve as the by-product of correlational selection in color polymorphic species, even if competition for resources is not severe. Moreover, color in animals may influence several aspects of performance and fitness (Forsman et al. 2008). In ectothermic animals, such as reptiles and insects that rely on external radiant energy for body temperature regulation, coloration may influence heating rates and body temperature as well as habitat use (Brown and Benson 1974; Darst and Cummings 2006; Gray and Mckinnon 2007; Nokelainen et al. 2012). Therefore, when alternative color morphs coexist in a population, correlational selection may result in modifications in genetic architecture and developmental pathways and lead to divergence among color morphs in morphology, physiology, behavior, and life-history traits (Forsman and Appelqvist 1998; Forsman et al. 2002; Svensson et al. 2008; Merrill et al. 2011). For example, individuals with either dark or pale coloration may need to seek out microhabitats that differ in thermal properties or be active at different time periods to maintain favorable body temperatures. In addition, they may use visually differing patches to camouflage and obtain protection from predators (Forsman et al. 2002; Ahnesjö and Forsman 2003). The protective value of color that functions as an avoidance-inducing signal may depend upon habitat choice, behavior, movement patterns, or body size (Gray and Mckinnon 2007). Therefore, different morphs are expected to use different subsets of available resources and occupy different niches on an average, suggesting that color polymorphism can lead to resource partitioning. In these cases, resource partitioning may evolve independent of resource competition. However, such processes have not been well tested. In this study, we examined resource partitioning in sexually selected female-limited color polymorphism in the damselfly *Ischnura senegalensis* in view of adaptive trait complexes relating to resource partitioning such as resource preference, utilization efficiency, and morphologies.

## Materials and Methods

### Study species and study sites

**Figure 1:**
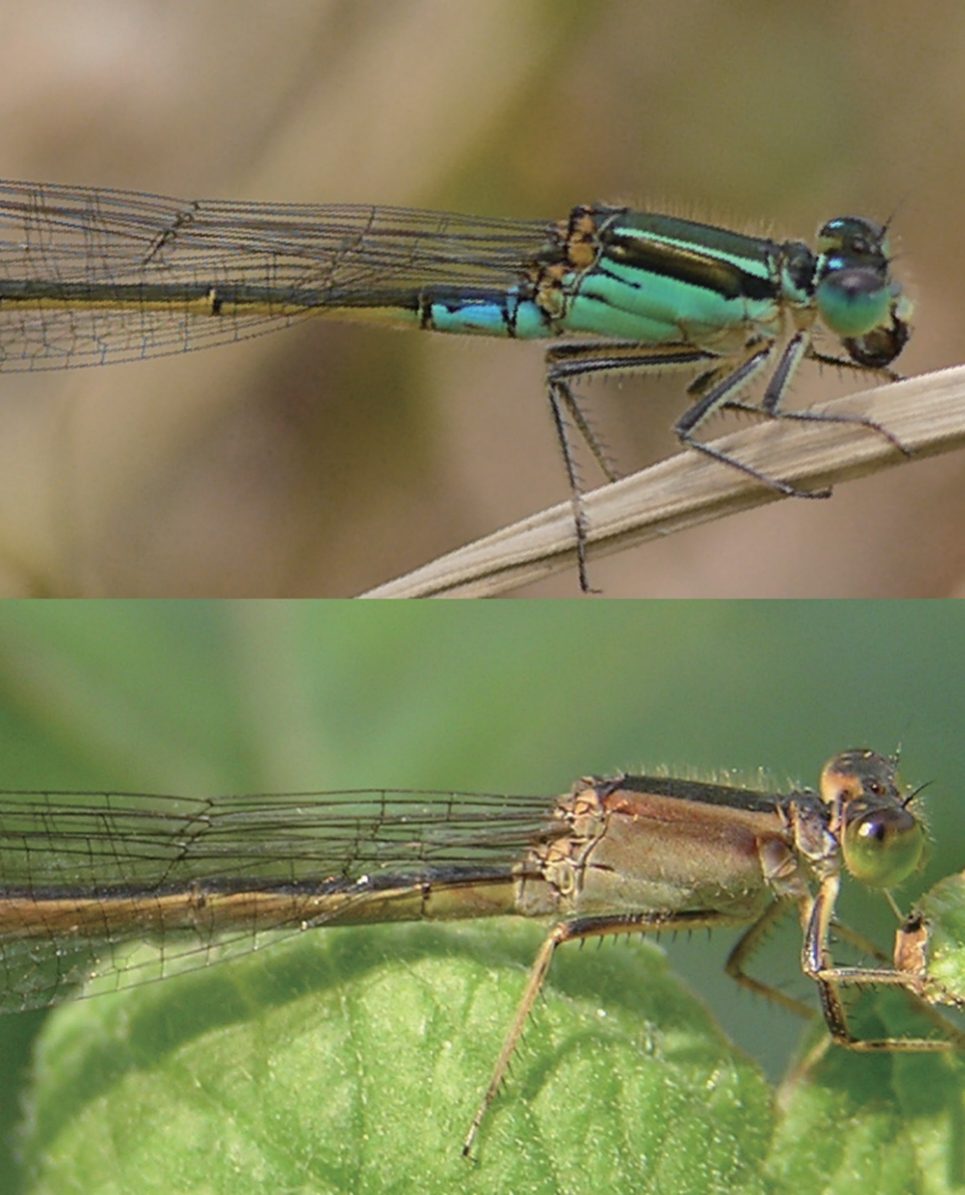
Female color polymorphism in common bluetail, *Ischnura senegalensis*. Andromorphs (top panel) have greenish thorax and gynomorphs (bottom panel) have brownish one.

*Ischnura senegalensis* is a nonterritorial damselfly. Females exhibit color dimorphism (Fig. 1), appearing as blue-green andromorphs and brown gynomorphs, although males have conspicuous green thoracic coloration (Corbet 1999; Gosden and Svensson 2009; Takahashi et al. 2012). Both males and females live in grasslands near ponds throughout their life spans, where they feed, mate, oviposit, and roost. Sexually mature males actively move about on the wing throughout the day searching for mates (Robertson 1985; Takahashi and Watanabe 2009).

We chose two local populations of *I. senegalensis* in Fukui Prefecture (Ozutsumi: 36.229623°N, 136.179848°E) and Iabaraki Prefecutre (Tsukuba: 36.157835°N, 140.063784°E), where the level of two female morphs was not extreme (30–70% of females were andromorphs) (Takahashi et al. 2011). In both habitats, the aquatic plants *Phragmites australis, Scirpus triangulates*, *Typha* spp., *Trapa japonica*, *Nymphaea* spp. (only in Ozutsumi), and *Egeria densa* (only in Tsukuba) provided oviposition resources for *I. senegalensis*.

### Morphological traits

Females of both morphs were sampled by netting from the two populations (Ozutsumi: June 2009, *N*_andromorph_ = 40, *N*_gnomorph_ = 40; Tsukuba: June 2011, *N*_andromorph_ = 15, *N*_gnomorph_ = 21). All individuals, including both immature and mature females, were taken to a laboratory. The lengths of the abdomen and hind wing in the captured females were measured using a slide caliper. Mature females were dissected under a stereomicroscope, and the length and width of mature eggs were measured with a micrometer for three eggs randomly sampled from each female. The volume (*V*) of the mature egg was subsequently calculated as a spheroid (*V* = *L* × *W*^2^×π/6), where *L* and *W* are the average length and width of the mature egg, respectively. Egg shape (*S*) was evaluated using the formula *S* = *L*/*W*. The length of the ovipositor was measured with a micrometer for females captured in Tsukuba (the ovipositor of females captured in Ozutsumi had not been observed due to the disposal of specimens).

### Observation of oviposition behavior

In the two populations, oviposition behaviors were observed in 2009 and 2011. We made behavioral observations from 1200 h, when females begin to lay eggs, to 1600 h, when oviposition activity declines, using focal animal methods (Altmann 1974). Females at the edge of a pond were observed from the shoreline by naked eye for as long as possible (average ca. 8.5 min), and their oviposition behaviors were observed. Because females laid eggs into the fragmented oviposition resources on the water surface, we recorded the species and status (fresh or decaying) of the plants into which a focal female attempted to insert her ovipositor, and the number of successful egg insertions into plant tissue in each oviposition trial based on the motions of the female abdomen; females pressed their abdomen against plant tissues after successful insertion of the ovipositor but not when failed (Corbet 1999). We chose the areas with mixed oviposition resources along the water's edge as the oviposition site (only in population Tsukuba). A pair consisting of an andromorph and a gynomorph was sequentially observed in the same area to eliminate the effect of vegetation on oviposition site choice. Observation areas in each population were at least 15 m away from each other to prevent sampling the same females multiple times accidentally. Because only *Nymphaea* spp. was inhabited in the area where we can observe females in the Ozutsumi population, data from this population were not used for the analyses of preference in oviposition resources. A total of 150 oviposition trials were observed using 7 andromorphs and 7 gynomorphs in Tsukuba and 57 oviposition trials using 3 andromorphs and 3 gynomorphs were observed in Ozutusumi.

### Statistical analyses

Statistical analyses were performed using R version 2.14.0 (R Development Core 2011). Egg morphology, body size (the length of abdomen and hind wing), and the length of the ovipositor were analyzed by two-way ANOVA with morph and population as factor. To eliminate the potential effects of pseudoreplication, the differences between morphs in the composition of plants used by females and in the number of eggs laid in each oviposition trial were analyzed by generalized linear mixed model (GLMM), assuming binomial and Poisson distribution, respectively. Individuals were treated as a random effect in GLMM because oviposition trials were counted multiple times in the same female, and the effect of year was included as factor. All analyses were two tailed. All values are presented as mean ± SE.

## Results

### Morphological traits

**Figure 2:**
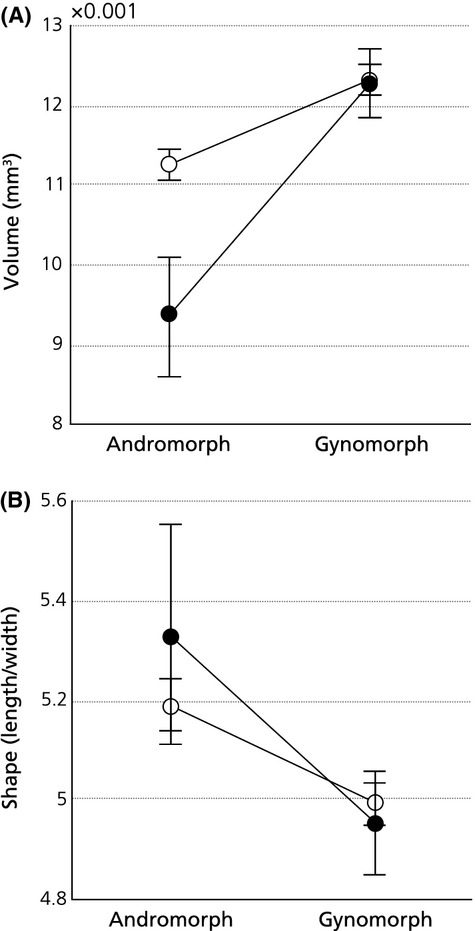
Egg morphology of each color morph (mean ± SE) (A) The volume of mature eggs oviposited. (B) Egg shape, which was calculated as length/width. Populations: Tsukuba is denoted by open circles and Ozutsumi as filled circles. The length of mature eggs for andromorphs in population Ozutsumi and Tsukuba was 0.831 ± 0.004 mm (*N* = 39) and 0.791 ± 0.006 mm (*N* = 5), respectively, and that for gynomorphs was 0.835 ± 0.004 mm (*N* = 40) and 0.829 ± 0.005 mm (*N* = 9), respectively. Likewise, the width of mature eggs for andromorphs in these two populations was 0.161 ± 0.001 mm (*N* = 39) and 0.150 ± 0.006 mm (*N* = 5), respectively, and that for gynomorphs was 0.168 ± 0.001 mm (*N* = 40) and 0.168 ± 0.003 mm (*N* = 9), respectively.

**Figure 3:**
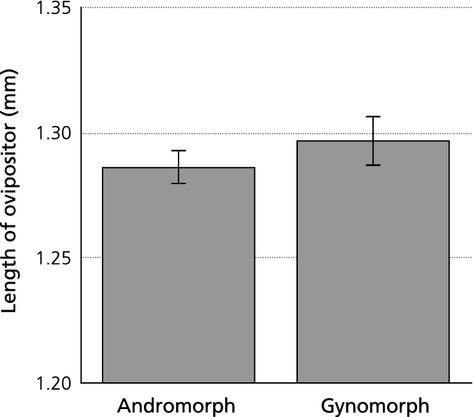
Length of ovipositor of each color morph (mean ± SE).

Average abdomen lengths in andromorphs (Ozutsumi: 27.26 ± 0.14, *N* = 40; Tsukuba: 26.50 ± 0.19, *N* = 15) were significantly larger than those in gynomorphs (Ozutsumi: 26.95 ± 0.13, *N* = 40; Tsukuba: 26.10 ± 0.13, *N* = 21), and significantly differed between populations (morph: *F* = 6.958, *P* = 0.0095; population: *F* = 24.36, *P* < 0.0001, interaction: *F* = 0.07, *P* = 0.79685). The estimated egg volume in andromorphs was significantly smaller than that in gynomorphs, and significant interaction effects of morph and population were detected (morph: *F* = 23.93, *P* < 0.0001; population: *F* = 3.95, *P* = 0.050; interaction: *F* = 5.48, *P* = 0.022) (Fig. 2). Egg shape index (*S*) in andromorphs was significantly larger than in gynomorphs, indicating that andromorphs have elongated eggs in their ovaries and gynomorphs have stubbier eggs (morph: *F* = 11.08, *P* = 0.0013; population: *F* = 0.11, *P* = 0.745; interaction: *F* = 0.86, *P* = 0.355). No difference in the ovipositor size was observed between morphs (*F* = 0.79, *P* = 0.389) (Fig. 3).

### Preference for oviposition resources

**Figure 4:**
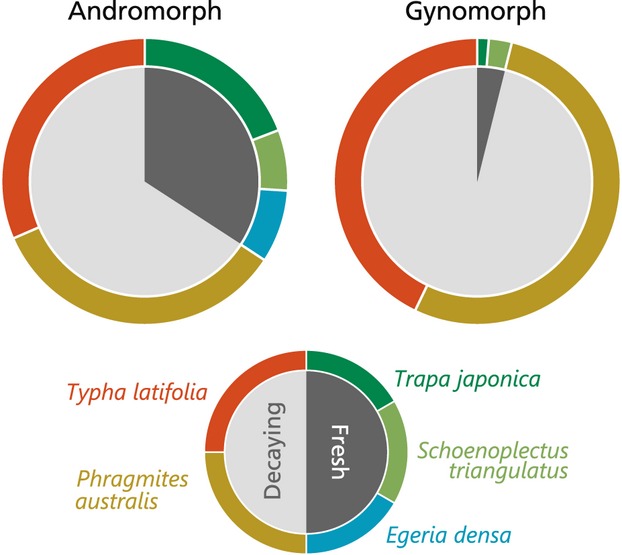
Preference for oviposition substrates. Substrates were divided into two categories: decaying tissues (*Typha latifolia*, *Phragmites australis*) and fresh tissues (*Trapa japonica*, *Egeria densa*, *Schoenoplectus triangulatus*).

Oviposition trials for the decaying tissue of *Phragmites australis* and *Scirpus triangulates* and the fresh (living) tissue of *Typha* spp., *Trapa japonica*, and *Egeria densa* were observed in Tsukuba (Fig. 4). The main oviposition resources were decaying plant tissues in both morphs, probably because of the abundance of decaying plant tissues on the water surface. The composition of these five tissues used as oviposition resources significantly differed between morphs (morph: *Z* = 2.65, *P* = 0.008; year: *Z* = 1.09, *P* = 0.274). The proportion of oviposition trials for fresh plant tissues in andromorphs was significantly higher than that in gynomorphs (morph: *Z* = −2.63, *P* = 0.006; year: *Z* = −1.09, *P* = 0.274).

### Utilization efficiency

**Figure 5:**
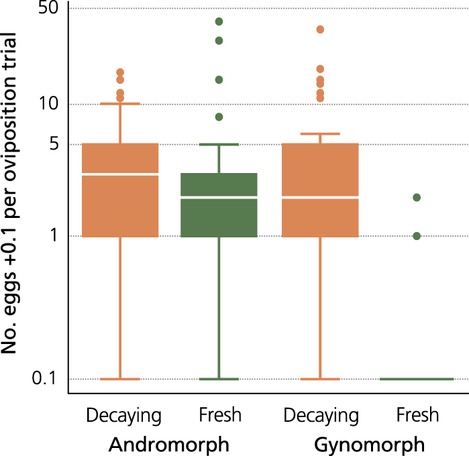
Oviposition efficiencies on each oviposition substrate. Substrates were divided into two categories: decaying tissues and fresh tissues (see Fig. 4 in detail).

Females very often altered oviposition location and laid eggs without competing for oviposition location with other females. The average number of ovipositor insertions into plant tissues per oviposition trial was 4.14 ± 0.49 (mean across 14 females [*N* = 150], range: 0–40). As shown in Fig. 5, both morphs successively inserted eggs into decaying plant tissues. However, almost all gynomorphs failed to insert eggs into fresh plant tissues, although andromorphs succeeded. Significant interaction effects were seen between the morph and the type of plant tissue (fresh or decaying) on the number of insertion per trial (morph: *Z* = −4.547, *P* < 0.0001; tissue: *Z* = −10.231, *P* < 0.0001; year: *Z* = 1.199, *P* = 0.231; population: *Z* = 3.512, *P* = 0.0005; morph × tissue: *Z* =5.425, *P* < 0.0001).

## Discussion

Disruptive selection induced by competition for limited resources is hypothesized to result in the evolution of resource partitioning (Rueffler et al. 2006). In other words, resource partitioning may not evolve when competition for resources is not severe. However, in this study, we describe resource partitioning in sexually selected color polymorphism in females of the damselfly *I. senegalensis*, for which oviposition resources (plant tissues) are sufficient. These resources are not depleted because females do not consume plant tissues during oviposition and competition for oviposition location was not observed in ovipositing females, suggesting that the resource partitioning in this species is not an evolutionary outcome of resource competition. In this system, we observed alternative adaptive preferences and egg morphologies that were associated with female body color. Correlational selection may result in secondary resource partitioning as a by-product of phenotypic integration with sexually selected body color, suggesting an overlooked mechanism leading to resource partitioning.

### Evolution of resource partitioning

The female damselflies change oviposition localities very often to reduce the risk of predation on the eggs by aquatic predators and cannibalism among siblings when they hatch, and the total oviposition lasts 1–3 h in *Ischnura* (Huang et al. 2012). Because the number of eggs laid daily is approximately 200 (Takahashi and Watanabe 2010a) and an average of four eggs are laid per oviposition trial (this study), females are estimated to alter their oviposition location at least 50 times a day. During each of the oviposition trial on the water surface and each of the oviposition site change between trials, females are exposed to various hazards: they are often harassed by males (Takahashi and Watanabe 2010a) and/or attacked by predators such as water spiders and water strider (Purse and Thompson 2009). Thus, selective use of plant tissues depending on body color must be adaptive, as in the cases of adaptive habitat use in other color polymorphic organisms (e.g., Shine et al. 2003). In this study, gynomorphs, which have a brown body, virtually used only brown (decaying) plant tissues, but andromorphs, which have blue-green bodies, laid eggs on green (fresh) plant tissues more frequently than gynomorphs, suggesting that they seek out oviposition substrate that provides most effective camouflage for their individual phenotype. Egg-laying substrate selection for optimal camouflage is reported in some animals (Lovell et al. 2013).

However, in general, behavioral adaptation is not enough to achieve utilizing different resources efficiently as well as typical trophic polymorphism; different morphs using alternative resources should have alternative morphological traits relating to resource use (Meyer 1989; Ruzzante et al. 1998). In Odonata, egg morphology correlates well with the oviposition pattern in dragonflies and damselflies, where species that lay eggs into plant tissues have elongated eggs (Corbet 1999), implying that injecting eggs is likely to be easier with more elongated eggs (Joop et al. 2007). In this study, we observed more elongated eggs in andromorphs that use fresh and hard plant tissues. Narrower eggs of andromorphs than those of gynomorphs have also been suggested in other populations and laboratory-reared individuals (Takahashi and Watanabe 2010c). This suggests that elongated eggs have evolved in andromorphs to improve oviposition in fresh (hard) plant tissues. The efficient oviposition in fresh plant tissues by andromorphs could be enhanced by the different egg morphologies. Therefore, preferences for plant tissues and egg morphology may be physically integrated with the body color of females. Correlational selection originates and maintains the adaptive combination of traits within each morph. Therefore, resource partitioning in the current system may be the by-product of an integration of morphological and behavioral differences with the color morphs. The difference in egg volume between two populations may result from a difference in body size.

### Genetic basis and maintenance of resource partitioning

In this study, we observed a phenotypic correlation between body color, resource preference, and egg morphology. Correlational selection favors the adaptive correlation of multiple traits governed by different loci. However, recombination and segregation collapse linkage disequilibrium, the nonrandom association of alleles at different loci, within a few generations depending on recombination frequency (Slatkin 2008). Thus, physical linkage of loci on the genome or assortative mating is important for maintaining linkage disequilibrium (Merrill et al. 2011). If the distance between loci controlling different traits is short, a lower recombination rate at a region between the loci can maintain the linkage disequilibrium. Because egg size, egg shape, and oviposition site preference may be qualitative traits governed by polygenes, one of loci affecting these traits may physically link with the color locus. On the other hand, assortative mating can potentially contribute to linkage disequilibrium even when multiple loci do not physically link to each other. In damselflies, males have the gene that controls female color morphs, although it does not affect male body color. Thus, if males having an andromorphic allele preferentially mate with an andromorph, and vice versa, as reported in *Ischnura* (Johnson 1975), the adaptive allele combination at different loci can be maintained in each morph. However, in the previous study, males have no innate preference for female morphs and they reversibly switch their preference depending on day-to-day mating experiences (Takahashi and Watanabe 2008, 2009, 2010b), suggesting that assortative mating, which maintains alternative genetic correlation is not active.

Alternatively, the integration of multiple phenotypic traits could be caused by the pleiotropic effects of a supergene (Joron et al. 2011). In this case, genes governing hormonal regulation are likely candidates to be major effect genes, owing to the potential for pleiotropic effects of hormones on color, behavior, physiology, and life-history traits (Ketterson and Nolan 1992; Sinervo et al. 2001; Svensson et al. 2002). In female polymorphic damselflies, phenotypic differences between the morphs have been suggested for behavioral responses to mating attempts by males (Robertson 1985; Sirot and Brockmann 2001), microhabitat selection by adults (Van Gossum et al. 2001), oviposition site preference (this study), the dispersal ability of adults (Svensson and Abbott 2005), fecundity (Gosden and Svensson 2007), egg size (Takahashi and Watanabe 2010c), egg shape (Joop et al. 2007), developmental response (Takahashi et al. 2011), larval developmental rate (Abbott and Svensson 2005; Abbott 2013), and the body shape of adults (Abbott and Gosden 2009; Abbott and Svensson 2010). Several of the morphological traits were heritable, and the morphologies, behaviors, and habitat choice of andromorphs were often similar to the males (Robertson 1985; Van Gossum et al. 2001; Abbott and Svensson 2010). This suggests that the gene for determining male characteristics leads to intermorph differences as supergene. However, at present we cannot distinguish between physical linkage, assortative mating, and the supergene (pleiotropic effect) in several qualitative traits. Further researches, such as developmental analyses, genomic analyses, and population genetic analyses, are necessary to identify the mechanism(s) producing phenotypic correlation in *I. senegalensis*.

### Ecological and evolutionary consequences

The coexistence of multiple trophic morphs is predicted to result in some ecological and evolutionary consequences. In general, the evolution of trophic polymorphisms may reduce intraspecific competition in cases of resource partitioning between species (Kornfield and Taylor 1983; McLaughlin et al. 1999). Therefore, different trophic morphs are expected to be associated with the utilization of broader niches by the population as a whole (Smith and Skúlason 1996). Thus, the evolution of a trophic polymorphism could enhance population performance, such as increasing the rate and carrying capacity (Ellers et al. 2011; Jousset et al. 2011). However, in this study, enhancement of population performance is not expected because the competition for oviposition resources was not severe. Therefore, the impact of resource partitioning on ecological dynamics may be negligible in trophic polymorphism as a by-product of sexual selection.

In addition, resource partitioning may affect evolutionary processes, such as speciation and gene-frequency dynamics. Divergence in a resource or habitat utilization often leads to rapid speciation (Kawata et al. 2007; Seehausen et al. 2008). When intermediate phenotypes cannot utilize resources efficiently and have disadvantages for resource competition, assortative mating, where individuals with similar genotypes selectively mate with one another, can evolve and is believed to be the cause of reproductive isolation. In general, resource partitioning with assortative mating might trigger speciation (Dieckmann and Doebeli 1999). However, the current resource partitioning in *I. senegalensis* may not directly lead to speciation due to the lack of assortative mating (Takahashi and Watanabe 2009, 2010b).

We observed that the size of mature eggs differed between the morphs as well as the egg shape, and that gynomorphs laid larger eggs than andromorphs. In Odonata, the size of early instar larvae depends on egg size (Schenk and Sondgerath 2005). The egg developmental time also depends on egg size because of its vitelline content (Hottenbacher and Koch 2006). These observations suggest that the gynomorphs offsprings grow faster and generate larger larvae than those of andromorphs. In addition, because size-dependent cannibalism during the early larval stages is commonly observed in Odonata in aquatic environments, smaller larvae are apt to be eaten by larger ones (Anholt 1994; Padeffke and Suhling 2003). Therefore, early instar larvae derived from gynomorphs must have higher survival rates than those from andromorphs. Furthermore, we observed that the oviposition efficiency using fresh plant tissue differed between morphs because of a difference in egg shape, suggesting that the reproductive success of each morph is partly affected by the composition of the oviposition substrates in each habitat. Thus, the differences in size and shape of eggs may potentially affect the overall fitness of each female morph, and thus be key factors in affecting the spatiotemporal dynamics of morph frequency in nature.
